# A Unique Synthesis of Macroporous N-Doped Carbon Composite Catalyst for Oxygen Reduction Reaction

**DOI:** 10.3390/nano11010043

**Published:** 2020-12-26

**Authors:** Ramesh Karunagaran, Diana Tran, Tran Thanh Tung, Cameron Shearer, Dusan Losic

**Affiliations:** 1 ARC Graphene Research Hub, School of Chemical Engineering and Advanced Materials, University of Adelaide, Adelaide, SA 5005, Australia; ramesh.karunagaran@adelaide.edu.au (R.K.); diana.tran@adelaide.edu.au (D.T.); 2 Institute of Research and Development, Duy Tan University, Da Nang 550000, Vietnam; 3 School of Chemistry, University of Adelaide, Adelaide, SA 5005, Australia; cameron.shearer@adelaide.edu.au

**Keywords:** porous carbon, N-doped carbon, electrocatalyst, carbon composites, sulphonated aniline

## Abstract

Macroporous carbon materials (MCMs) are used extensively for many electrocatalytic applications, particularly as catalysts for oxygen reduction reactions (ORRs)—for example, in fuel cells. However, complex processes are currently required for synthesis of MCMs. We present a rapid and facile synthetic approach to produce tailored MCMs efficiently via pyrolysis of sulfonated aniline oligomers (SAOs). Thermal decomposition of SAO releases SO_2_ gas which acts as a blowing agent to form the macroporous structures. This process was used to synthesise three specifically tailored nitrogen (N)-doped MCM catalysts: N-SAO, N-SAO (phenol formaldehyde) (PF) and N-SAO-reduced graphene oxide (rGO). Analysis using Scanning Electron Microscopy (SEM), Fourier Transform Infrared Spectroscopy (FTIR) and X-ray diffraction (XRD) analysis confirmed the formation of macropores (100–350 µm). Investigation of ORR efficacy showed that N-SAOPF performed with the highest onset potential of 0.98 V (vs. RHE) and N-SAOrGO showed the highest limiting current density of 7.89 mAcm^−2^. The macroporous structure and ORR efficacy of the MCM catalysts synthesised using this novel process suggest that this method can be used to streamline MCM production while enabling the formation of composite materials that can be tailored for greater efficiency in many applications.

## 1. Introduction

Macroporous carbon materials (MCMs) can be classified as carbonaceous materials with pore sizes >50 nm [1]. These materials are in great demand for many applications due to their large surface area, physiochemical stability, good conductivity and low cost [2]. In particular, these materials are widely used in supercapacitors [3], sensing technologies [4], as adsorbents for herbicides [5] and CO_2_ [6], as well as a catalyst for reactions involving the reduction of oxygen (O_2_) [7] and nitric oxide (NO) [8]. Carbonaceous materials with larger pore sizes have been found to be effective catalysts because they facilitate electrolyte permeability, efficient mass transport and diffusion of reactants and by-products [9]. Since most catalyst active sites are deeply rooted in the porous catalyst structure, macropores enable greater access to these active sites [10], thus enhancing electrocatalytic performance. Previous studies have revealed that electrocatalytic performance increases with an increasing pore size. As such, porous carbon catalysts with macropores (150 nm) have demonstrated greater efficacy in oxygen reduction reactions (ORRs) than catalysts with mesopores (12 nm) [11] and, likewise, mesoporous (14 nm) catalysts have outperformed those with micropores (1 nm) [12].

Synthesis of MCMs is possible using several methods, most commonly with a template method that uses anodic aluminium oxide (AAO), spherical submicron sized silica particles, aluminium impregnated silica particles and polystyrene (PS) microspheres [13]. Other methods include a sol-gel process which requires lengthy crystallisation steps [14]; chemical vapour deposition where toxic organic solvents such as benzene are super-heated [15]; metal organic frameworks (MOFs) which need lengthy synthesis procedures to achieve a low yield [16]; or decomposition methods that need complicated apparatus [17]. These procedures are limited due to the sophisticated process methodology and extended steps of the synthesis process. Furthermore, the commonly used template method requires the removal of templates after synthesis, which requires the use of highly hazardous materials, such as hydrogen fluoride (HF) [13]. Therefore, novel synthesis methods that are less laborious, facile and reduce the need for toxic solvents would be of great benefit for MCM production. 

To perform a smooth and rapid electrochemical reaction such as ORR in fuel cells, fast mass exchange of reactants and products is essential to enhance efficiency of the catalysts. MCMs that incorporate other materials can increase the catalytic efficacy for such applications. Nitrogen-doped (N-doped) macroporous carbon materials, in particular, are extensively utilised as catalyst materials for many applications including ORR [18] and in supercapacitors [19] and batteries [20,21]. Graphene, with its outstanding properties such as large specific theoretical surface area (2630 m^2^g^−1^) [22], high Young’s modulus (approx. 1.0 TPa) [23], high intrinsic mobility (200,000 cm^2^V^−1^s^−1^) [24] and thermal conductivity (approx. 5000 Wm^−1^K^−1^) [25] has provided mechanical stability and increased charge transfer that can also increase catalytic reaction efficacy [26,27]. N-doped macroporous carbon materials have been synthesised by adopting several approaches, including pyrolysis of biomass [28], hydrothermal treatment of glucose [29], ternary doping of carbon fibres [30], and template methods [18]. Incorporation of graphene in MCMs is commonly achieved by thermal reduction of graphene oxide (GO) to reduced graphene oxide (rGO) [31]. Effective methods that can readily synthesise N-doped MCM or graphene-doped MCM would enable the rapid production of efficient catalytic materials for several applications, including ORR for fuel cells.

Synthesis of the large pores (bigger than 50 nm) of MCM is an important and difficult stage of producing this material. Traditionally, a blowing agent—such as carbon dioxide, nitrogen or hydrogen peroxide—must be added to the reaction to increase the pore size to create MCMs [32,33]. However, the use of sulphur dioxide (SO_2_) as a blowing agent has been not commonly reported. A study conducted on polycondensation of poly(l-lactic acid) using aromatic sulfonic acid determined that sulphonic acid thermally decomposed in the temperature range of 200–300 °C [34]. The decomposition product, SO_2_, is a potential blowing agent used in MCM production. Therefore, a base material that incorporates aromatic sulphonic acid, such as sulphonated aniline oligomers (SAOs), may enable effective MCM production. SAOs are readily synthesised using excess aniline and oxidising agent, ammonium persulphate, in an acidic medium [35]. The monomer units rearrange to form sulfonated ortho semidine and para semidine with a sulphonic group (SO_3_H) attached to the aromatic ring. SAO is an organic base, and the pH of the medium can alter its surface chemistry and reaction kinetics to form diverse structures including spheres, sheets, flakes, rods and granules [36]. The sulphonic groups attached to SAO can potentially decompose during pyrolysis to form SO_2_ gas that could contribute to MCM production.

In this study, we evaluated the efficacy of SAO in the production of tailored MCMs using in situ released SO_2_ as a blowing agent. Using this technique, we synthesised an N-doped carbon catalyst by the pyrolysis process of SAO with a nitrogen precursor melamine (N-SAO). Furthermore, we synthesised two more N-doped composite materials of SAO with reduced graphene oxide (N-SAOrGO) and phenol formaldehyde (PF) (N-SAOPF) to introduce diverse active sites for oxygen adsorption in ORR reactions. The structure of each material was characterised and the efficacy of each as an ORR catalyst was determined.

## 2. Experimental

### 2.1. Materials

Natural graphitic rock (Uley, Eyre Peninsula, South Australia, Australia) was milled into a fine powder using a bench top ring mill (Auckland, New Zealand). Ammonium persulphate ((NH_4_)_2_S_2_O_8,_ APS), aniline (Reagent Plus, 99%), ethanol, hydrogen peroxide (H_2_O_2_), hydrochloric acid (HCl), melamine, potassium permanganate (KMnO_4_), phenol, paraformaldehyde, phosphoric acid (H_3_PO_4_) and sulphuric acid (H_2_SO_4_) were purchased from Sigma-Aldrich (Australia) and Chem Supply (Australia). Nafion 10 wt.% in water solution and platinum standard catalyst (20% Vulcan XC-72) were purchased from Fuel Cell Store (College Station, TX, USA).

### 2.2. Methods

#### 2.2.1. Preparation of SAO and SAOrGO, SAOPF and rGOPF Composites

##### Preparation of SAO

The SAO was synthesised using a previously published method [35]. Aniline (5 mL, 5.19 g, 0.05 mol) was dissolved in 40 mL of ethanol. Subsequently, APS (1.35 g, 0.005 mol) dissolved in 6 mL of 1 M HCl was slowly added. The solutions were mixed at 5 °C for 30 min using a magnetic stirrer and then continued stirring at room temperature for 16 h. To purify, the product was then centrifuged at 4200 rpm in a centrifuge. The precipitate was collected and continuously washed with ethanol for several hours in a Soxhlet extractor and vacuum dried for overnight.

##### Preparation of Graphene Oxide (GO) and SAOrGO Composites

*Preparation of GO*

GO was synthesised using the improved Hummer’s method [37]. A mixture of concentrated acids H_2_SO_4_/H_3_PO_4_ (9:1) was added to a mixture of graphite powder (3.0 g) and KMnO_4_ (18.0 g). The solution was then heated to 50 °C and stirred for 12 h. The reaction was cooled and poured onto ice (400 mL) containing H_2_O_2_ (3 mL). The graphite oxide was purified by centrifuging at 4200 rpm for 1 h and the supernatant was removed. The precipitate was then washed several times with water, and then 30% HCl (2 times of 200 mL) and finally with ethanol (2 times of 200 mL). The final product was collected, and vacuum dried overnight at room temperature.

*Preparation of SAOrGO composite*

GO (30 mg) was added in 15 mL of distilled water and sonicated for 1 h. The GO suspension was diluted with 15 mL ethanol and transferred into a Teflon autoclave and heated in an oven for 90 min at 150 °C. The autoclave was cooled down in ambient, and 5 mL of the hydrothermally reduced GO (rGO) was collected and mixed with aniline (5 mL, 5.19 g, 0.055 mol) dissolved in 40 mL of ethanol for 10 min. Then, APS (1.35 g, 0.005 mol) dissolved in 6 mL of 1M HCl was added. The dispersion was mixed at 5 °C for 30 min using a magnetic stirrer and then continued stirring at room temperature for 16 h. The product was then collected and centrifuged at 4200 rpm in a centrifuge. The precipitate was collected and continuously washed with ethanol for several hours in a Soxhlet extractor and vacuum dried for overnight.

##### Preparation of PF and SAOPF Composites

*Preparation of PF*

PF was synthesised using a minor modification to the procedure suggested by Meng et al. [38]. Briefly, phenol (0.22 mol) was melted for 10 min at 35 °C. Then, NaOH (0.1 mol) was dissolved in 12 mL of distilled water and mixed with the melted phenol and stirred in a magnetic stirrer for 5 min. Then, formaldehyde (para formaldehyde (10.40 g) in 28 mL distilled water) was added dropwise to the phenol suspension and mixed for 25 min at 80 °C until a transparent orange colour was obtained. Finally, the suspension was cooled, and the pH was adjusted to 3 by adding concentrated HCl solution. The product was allowed to settle overnight and the PF resin (10 mL) was collected and diluted with 5 mL of ethanol to prepare the PF resin solution.

*Preparation of SAOPF composite*

PF resin solution (7.5 mL) was mixed 12.5 mL of ethanol and 15 mL of distilled water and hydrothermally heated for 90 min at 150 °C in a Teflon autoclave. The hydrothermally heated product was cooled, and 5 mL of the hydrothermally treated resin solution was collected and mixed with aniline (5 mL, 5.19 g, 0.055 mol) dissolved in 40 mL of ethanol for 10 min. Then, APS (1.35 g, 0.005 mol) separately dissolved in 6 mL of 1M HCL, was added. The rest of the experimental procedure was conducted similarly to that of synthesis of SAO.

*Preparation of rGOPF composites*

The rGO/PF was prepared using a hydrothermally synthesised rGO/PF suspension. The suspension (5 mL) was mixed with the oxidising agent APS (1.35 g, 0.005 mol) which was dissolved in 6 mL of 1M HCl. The experimental procedure was carried out using a similar procedure as per SAO without using the monomer aniline.

#### 2.2.2. Preparation of MCM Composites (P-SAO, P-SAOrGO, P-SAOPF, and P-rGOPF)

*Preparation of pyrolyzed MCM composites*

SAO, SAOrGO, SAOPF and rGOPF (1.0 g) were placed on ceramic boats and separately pyrolysed in a tubular furnace at 900 °C for 30 min under argon environment, with a temperature ramp rate of 10 °C/min. The annealed products were referred to as P-SAO, P-SAOrGO, P-SAOPF and P-rGOPF, respectively. 

#### 2.2.3. Preparation of N-doped MCM Composites (N-SAO, N-SAOrGO, N-SAOPF and N-rGOPF)

SAO, SAOrGO, SAOPF and rGOPF (1.0 g) were separately mixed with melamine (1:10 *w*/*w*) using a mortar/pestle, and separately pyrolysed in a tubular furnace at 900 °C for 30 min under argon with a temperature ramp rate of 10 °C/min. The N-doped products were referred as N-SAO, N-SAOrGO, N-SAOPF and N-rGOPF, respectively.

### 2.3. Preparation of Catalytic Ink

Nafion solution (1 mL) was diluted with 10 mL with distilled water to obtain 1%wt. Nafion in water. Catalytic ink was prepared by ultrasonication of each catalyst (2 mg) in a 1 mL of diluted Nafion suspension. The prepared ink (10 µL) was carefully deposited on both a glassy carbon rotating disc electrode (3 mm) and rotating ring disc electrode (4 mm). The sample was allowed to dry in air for 12 h.

### 2.4. Characterisation

#### 2.4.1. Structural and Chemical Composition Characterisation

Fourier transform infrared (FTIR) spectroscopy was completed using Spectrum 100, Perkin Elmer, USA. X-ray diffraction (XRD) analysis was conducted at 40 kV and 15 mA in the range of 2θ = 3–80° at a speed of 10°/min using Miniflex 600, Rigaku (Akishima, Japan). Transition electron microscopy (TEM) investigation was performed using Technai G2 Spirit, FEI (Hillsboro, OR, USA), operated at 120 keV. Scanning electron microscopy (SEM) images were measured using Quanta 450, FEI (Hillsboro, OR, USA) with an accelerating voltage of 10 keV. XPS was conducted on a custom-built SPECS instrument (Berlin, Germany). All XPS measurements were performed on sample prepared by drop casting onto Si substrate using a nonmonochromatic Mg source operating at 120 keV and 200 W. High resolution XPS spectra were collected using a pass energy of 10 eV with an energy step of 0.1 eV. Raman analysis was performed using a LabRAM Evolution, Horiba Jvon (Tokyo, Japan) using a 532 nm wavelength. The thermometric-mass spectrometer experiment was carried out using a custom-build high temperature pulsed-gas sampling equipment coupled with a mass spectrometer for real-time analysis of the gaseous mixture. The detailed procedure is presented in the supporting information (Scheme S1 in Supplementary Materials).

#### 2.4.2. Electrochemical Characterisation

The ORR reactions were conducted utilising a Rotating Ring Disc Electrode (RRDE) apparatus connected to a bipotentiostat (CH 1760 C, CH Instruments Inc., Austin, TX, USA) in a standard three-electrode cell with oxygen saturated KOH (0.1 mol L^−1^) solution. The glassy carbon electrode, platinum and reversible hydrogen electrode (RHE) were used as the working, counter and reference electrodes, respectively. The scan rate of the reaction was 0.01 Vs^−1^ in the range of 0 and 1.1 V vs. RHE. Cyclic voltammetry plots were obtained at a scan rate of 100 mVs^−1^ in oxygen saturated 0.1 M KOH solution.

The reaction kinetic of the catalysts was examined by employing RRDE to quantify the overall electron transfer number (*n*) and percentage of hydroperoxyl (% HO_2_^−^), at rotation speeds from 400 to 2400 rpm in an oxygen saturated 0.10 M KOH solution. To elucidate the overall number of electrons (*n*) and percentage of HO_2_^−^ (% HO_2_^−^) produced in the ring against the applied potential, Equations (1) and (2) were employed [39,40].

(1) $$n=\frac{4I_{D}}{I_{D}\;+\;\frac{I_{R}}{N}}$$

(2) $$\;\;\;{\;\%\mathrm{HO}}_{2}^{-}=100\;\frac{2I_{R}}{I_{D}N+\;I_{R}}$$

where *I_D_* and *I_R_* are the disc and ring currents, respectively, and *N* is the collection efficiency.

## 3. Results and Discussion

### 3.1. Structural Characterisation of SAO and P-SAO

**Scheme 1:**
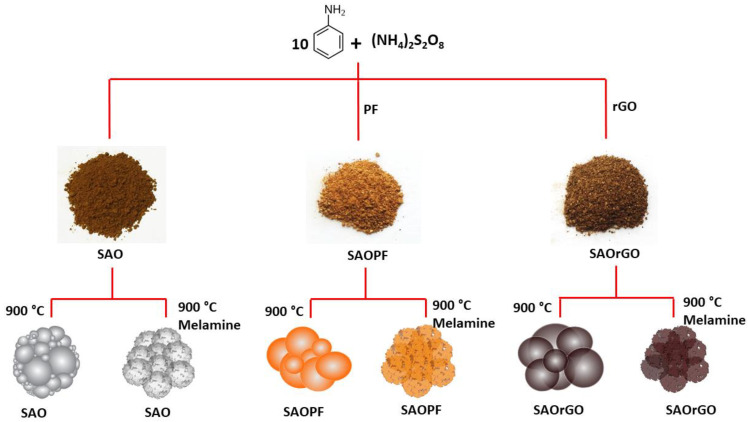
A schematic representation of the preparation of macroporous carbon materials (MCMs).

**Figure 1:**
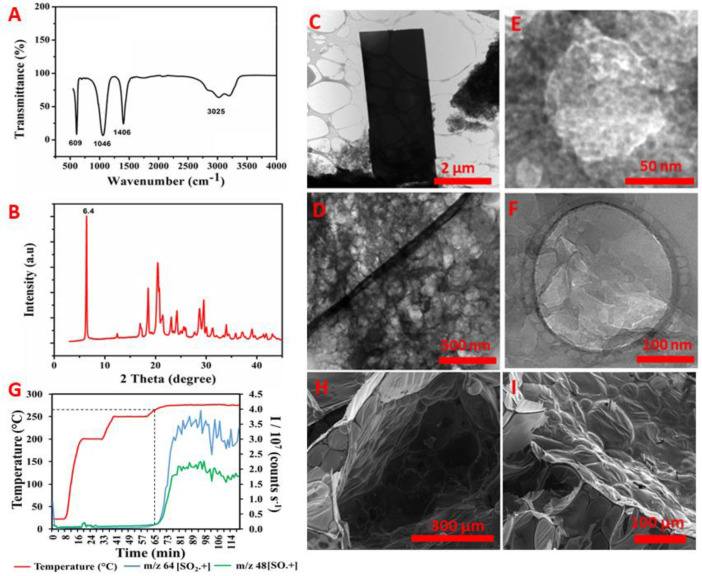
FTIR spectrum of (**A**) sulfonated aniline oligomers (SAOs), (**B**) XRD diffraction peaks of SAO. TEM images of: (**C**) SAO macroflake, (**D**) bunch of spherulites of the surface of macrosheets, (**E**) isolated single spherulites and (**F**) expansion of a spherulites. (**G**) The temperature-dependant mass spectrum of SAO in the temperature range of 30–275 °C, (**H**,**I**) “bubble wrap” type macroporous P-SAO.

The photographic image of a synthesised SAO is shown in Scheme 1. The structural characterisation of SAO was performed by FTIR and XRD. The FTIR spectrum of the SAO is shown in Figure 1A and shows distinct peaks at 609, 1046, 1406 and 3025 cm^−1^. The peak at 609 cm^−1^ corresponds to the vibration modes of the C-S bond [35,41]. The peak at 1046 cm^−1^ is ascribed to the characteristic peak of S=O stretching vibration mode of sulphonic groups linked to the benzene ring [35,41]. This clearly shows the presence of sulphonic groups in SAO. The peaks at 1406 and 3025 cm^−1^ correspond to the symmetrical stretching of phenazine heterocyclic rings and C-H aromatic stretching vibrations, respectively [35,42]. The XRD diffractogram of SAO (Figure 1B) shows an intense peak at 2θ = 6.4°, which is the characteristic peak for periodically aligned lamella of SAO chains [43,44]. The XRD analysis confirms that the lamellae of SAO chains are periodically arranged.

**Scheme 2:**
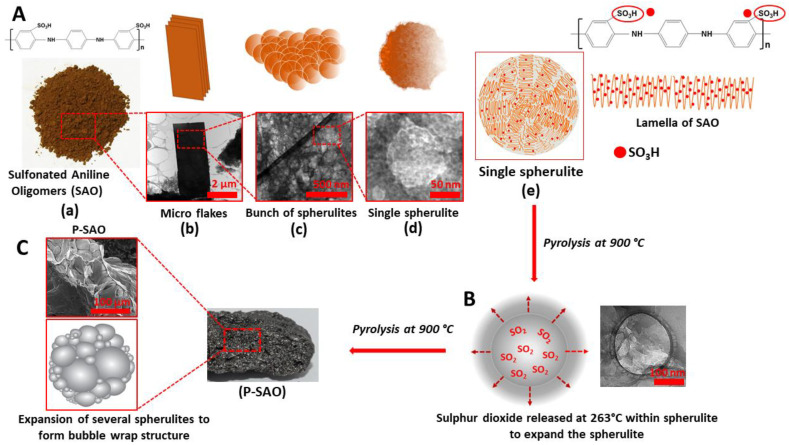
Schematic procedure of synthesis of microporous carbon material (P-SAO) using SAO. (**A**) Synthesised SAO, SAO macroflakes, spherulites on the flake surface, single isolated spherulite, a schematic representation of a spherulite; (**B**) expansion of spherulites; (**C**) macroporous P-SAO.

The TEM image of the SAO presented in Figure 1C and Scheme 2A(b) shows that the SAO consists of macrosize sheets. Interestingly, the higher magnification of these sheets revealed several nanosized (20–50 nm) spheres on the sheet surface of these sheets (Figure 1D and Scheme 2A(c). A magnified image of a single sphere is shown in Figure 1E and Scheme 2A(d). When polymers undergo crystallisation in the absence of a temperature gradient, the growth occurs radially with the linear polymer chains. These chains are folded and aligned together to form ordered regions called lamella (Scheme 2A(e)) [45]. The presence of these lamella in SAO is evident by the intense diffraction peak 2θ = 6.4° seen in Figure 1B. These lamellae with attached sulphonic groups have the tendency to arrange spherically to form spherulites (Figure 1E) [45]. A schematic representation of a spherulite is shown in Scheme 2A(e). The sulphonic group attached to the lamella can thermally decompose at higher temperatures [34]. A previous study conducted on the polycondensation of poly(l-lactic acid) using aromatic sulphonic acid determined that sulphonic acid thermally decomposes in the temperature range of 200–300 °C [34]. Therefore, we hypothesised that the sulphonate group attached to the polymer chain in the spherulites could decompose at an elevated temperature to release SO_2_ gas within the spherulites and expand them like air bubbles. To examine the release of SO_2_, we conducted a thermometric analysis on SAO using a custom-build high temperature pulsed-gas sampling equipment coupled with a mass spectrometer for real-time analysis of the gaseous fragments. The detailed experimental procedure is presented in the Supplementary Materials. The mass spectrometer detected the fragments of *m*/*z* 64 [SO_2_^.^+] and m/z 48 [SO^.^+] at 266 °C, confirming the release of SO_2_ (Figure 1G). Figure 1F and Scheme 2B show an expansion of a spherulite. We further hypothesised that the pyrolysis of SAO could produce MCM using SO_2_ gas as a blowing agent. A SEM image of the MCM synthesised by the pyrolysis of SAO under inert (argon) conditions (P-SAO) is presented in Figure 1H, I and Scheme 2C. The image shows a morphology of interconnected macropores with a pore size 100–350 µm.

### 3.2. Structural Characterisation of SAOrGO and SAOPF

**Figure 2:**
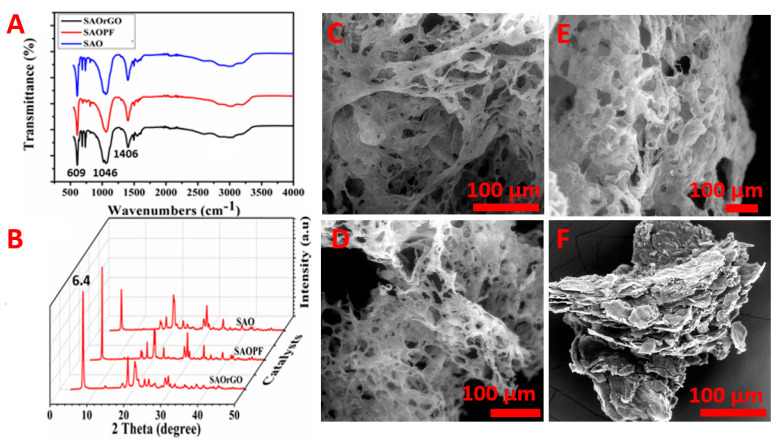
FTIR spectra of (**A**) SAO, SAOrGO and SAOPF; (**B**) XRD diffractograms of SAO, SAOrGO and SAOPF. SEM images of macroporous nitrogen doping catalysts: (**C**) N-SAO, (**D**) N-SAOrGO, (**E**) N-SAOPF and (**F**) N-rGOPF.

Using this unique synthesis process, two composites materials of SAO were synthesised using PF (SAOPF) and rGO (SAOrGO) and the photographic images of SAOPF and SAOrGO are shown in Scheme 1. The FTIR and XRD analyses conducted on these samples are presented in Figure 2A,B, respectively, revealing identical patterns similar to SAO. The pyrolysis of these products formed microporous structures similar to P-SAO. The SEM image of the pyrolysed SAOPF (P-SAOPF) and SAOrGO (P-SAOrGO) are shown in Figure S1 in the Supporting Information.

### 3.3. Structural Characterisation of N-SAO, N-SAOPE, N-SAOrGO and N-rGOPE Composites

The composite materials SAO, SAOPE, SAOrGO and rGOPF were pyrolysed with a nitrogen precursor melamine to synthesise N-doped MCM catalyst materials for ORR. The SEM images of N-SAO, N-SAOPE and N-SAOrGO are shown in Figure 2C–E. For a comparison study, we also synthesised a blend of rGO and PF without SAO (rGOPF, Figure 2F) to determine the effect of catalysts without the contribution of SAO. The blend of both rGO and PF was used for this study instead of individual rGO and PF because both rGO and PF under similar synthesis conditions did not provide enough yield. The SEM images revealed that all composite materials N-SAO, N-SAOrGO and N-SAOPF formed macroporous structures. However, such pores were not observed for rGOPF (Figure 2F), further confirming that these pores were formed from decomposition of SO_2_ gas from SAO.

**Figure 3:**
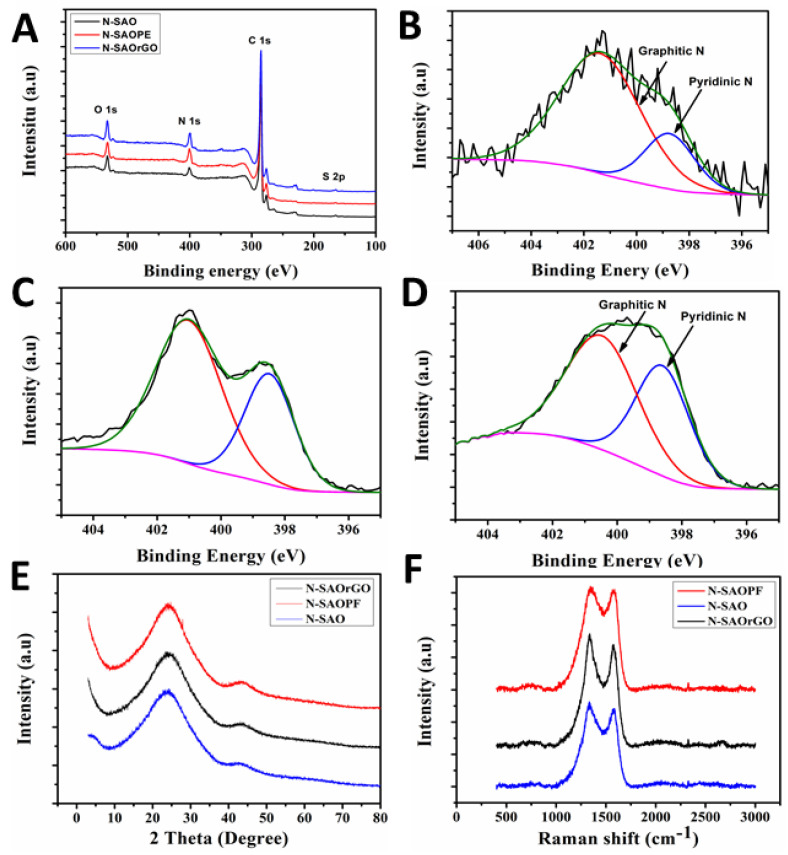
Survey spectrum of (**A**) N-SAO, N-SAOrGO and N-SAOPF and high resolution N1s XPS spectrum of (**B**) N-SAO, (**C**) N-SAOrGO, and (**D**) N-SAOPF. (**E**) XRD pattern and (**F**) Raman peaks of N-SAO, N-SAOrGO and N-SAOPF.

To investigate the presence of different chemical species present in N-SAO, N-SAOrGO and N-SAOPF, an XPS survey spectrum was conducted on these samples and is presented in Table 1 and Figure 3A. The survey spectrum shows a predominant C 1s peak at ca. 285.1 eV, N 1s at ca. 400.6 eV, O 1s peak at ca. 532.1 eV and a very low intensity S 2p peak at ca. 163.1 eV [46,47,48]. The presence of N1s peak reveals that the N-doping of the graphitic carbon structure has been established. N-SAO, N-SAOrGO and N-SAOPF revealed an atomic nitrogen percentage of 8.78, 4.86 and 8.89%, respectively.

The low intensity S 2p peak suggests the existence of small amounts of residual sulphur in the materials due to decomposition of sulphates. However, the atomic concentration of this peak is less than 0.80% in all samples.

To probe the different nitrogen species, present in the graphitic frame work, we deconvoluted high resolution N 1s measurements on N-SAO, N-SAOrGO and N-SAOPF and presented in Figure 3B–D. The high resolution XPS N 1s spectrum of these composites revealed the presence of two characteristic peaks of pyridinic (~398.4 eV) and graphitic (~401.3 eV) nitrogen atoms within the graphitic carbon structure. These pyridinic and graphitic nitrogen groups contribute differently towards ORR catalytic activity. Although it is still controversial, concerning the role of each of these active sites, it is widely believed that pyridinic nitrogen improves the onset potential while the graphitic nitrogen determines the limiting current density [49]. Pyridinic nitrogen alters the band structure of carbon by providing one *p* electron to the aromatic π system and raises the density of the π state closer to the Fermi level to enhance the electron-donating capability of the ORR activity. The pyridinic nitrogen induces high positive charge density on adjacent carbon atoms and increases the electron donor properties of these carbon atoms to adsorb O_2_. This diatomic oxygen adsorption on the carbon atom adjacent to pyridinic nitrogen weakens the O–O bond and facilitates the ORR reaction [49].

The comparison of the percentage atomic concentrations of these peaks (Table 2) reveals that the pyridinic nitrogen concentration of SAO composites (N-SAOrGO and N-SAOPF) is higher than that of N-SAO.

This shows that the addition of PF and rGO to SAO has influence the formation of pyridinic nitrogen in the carbon frame work. N-SAOPF showed the highest percentage (%) atomic concentration of pyridinic nitrogen (42.4%) while N-SAOrGO showed 36.69%. Furthermore, the results show that SAO facilitates more graphitic N substitution and less pyridinic N substitution.

XRD and Raman analyses were utilised to analyse the presence of graphitic carbon materials in the product. The XRD patterns (Figure 3E) and Raman analysis (Figure 3F) conducted on N-doped materials show that the materials are composed of graphitic carbon materials. The two distinctive XRD peaks at 2θ = 24.0 and 43.5 are attributed to the (002) reflection in turbostratic graphitic carbons and (101) Bragg reflection in graphitic carbon [50]. Raman analysis on the composite materials exhibits two peaks around 1340 and 1575 cm^−1^ corresponding to D band and G band of graphitic carbon. The graphitic peaks, G and D, arise due to the E_2g_ vibrational mode of the C–C bond stretching and the disorder peak due the A_1g_ vibrational mode. The intensity ratio of the D and G band (I_D_/I_G_) determine the defects associated with the pyrolysed and N-doped samples, where a higher intensity (I_D_/I_G_) ratio show more defects [51,52]. The calculated intensity ratios (I_D_/I_G_) showed 1.07, 1.08 and 1.02 for N-SAO, N-SAOrGO and N-SAOPF, respectively. The higher intensity ratio shows that reasonable distortion to the graphitic framework has taken place due to N-doping.

### 3.4. Electrochemical Characterisation of Catalytic Performances for ORR

**Figure 4:**
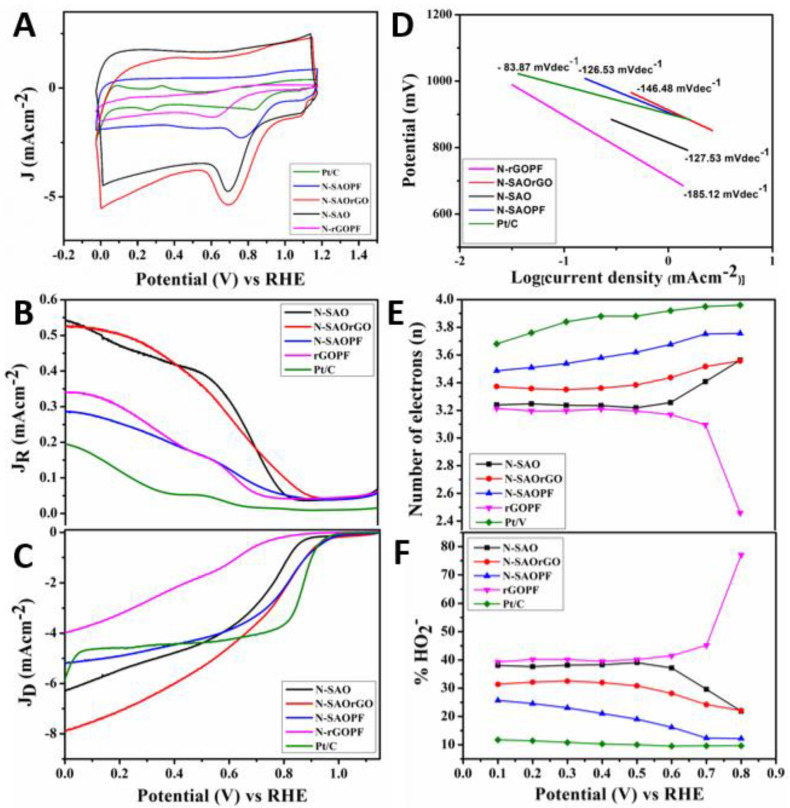
(**A**) Comparison of cyclic voltammetry plots of N-SAO, N-SAOrGO, N-SAOPF, N-rGOPF and Pt/C catalysts at a scan rate of 10 mVs^−1^ in oxygen saturated 0.10 M KOH solution. Rotating ring disc voltammograms of (**B**) ring current and (**C**) disc current of N-SAO, N-SAOrGO, N-SAOPF, N-rGOPF and Pt/C electrodes in oxygen saturated 0.1M KOH at 2000 rpm at a scan rate of 10 mV/s. (**D**) Tafel plots of the prepared catalysts: N-SAO, N-SAOrGO, N-SAOPF, N-rGOPF and Pt/C. (**E**) Number of electrons and (**F**) percentage peroxide of N-SAO, N-SAOrGO, N-SAOPF, N-rGOPF and Pt/C electrodes at various potential calculated according to Rotating Ring Disc Electrode (RRDE) data.

The electrochemical catalytic activity for ORR of N-doped (N-SAO, N-SAOrGO, N-SAOPF and N-rGOPF) catalysts were examined by cyclic voltammetry (CV) and presented in Figure 4A in an O_2_ saturated 0.10 M KOH solution in the potential range of 0.00 to 1.20 V vs. RHE. The voltammograms show that, for all catalysts, the CV curves displayed well defined oxygen reduction cathodic peaks centred at 0.67, 0.68, 0.76, 0.59 and 0.84 V, for N-SAO, N-SAOrGO, N-SAO, N-rGOPF and Pt/C, respectively, demonstrating catalytic activity for ORR. The ORR cathodic peak positively shifted in the order of Pt/C > N-SAOPF > N-SAOrGO > N-SAO > N-rGOPF. This reveals that the N-doped composite materials of SAO (N-SAOrGO and N-SAOPF) enhanced the catalytic activity of ORR as opposed to that of the N-SAO itself. It is interesting to note that the cathodic peaks shifted positively according to the increasing percentage of pyridinic nitrogen seen in Table 3.

**Figure 5:**
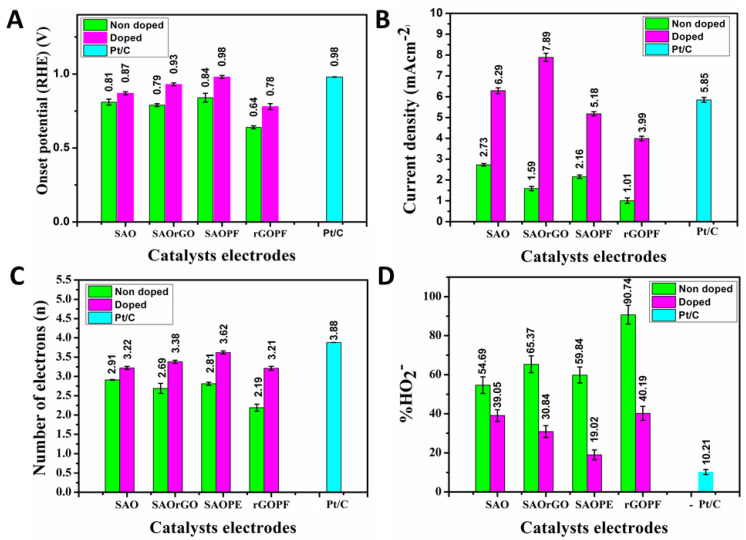
(**A**) Onset potential, (**B**) current density at 0.00 V (RHE), (**C**) comparison of number of electrons (at 0.50 V) and (**D**) % HO_2_^−^ (at 0.50 V) of nondoped and doped SAO, SAOrGO, SAOPF, rGOPF and Pt/C catalysts in oxygen saturated 0.10 M KOH electrolyte at 2000 rpm at a scan rate of 10 mV/s.

To quantify the ORR electron transfer pathway, we employed an RRDE technique to accurately measure the ring and disc currents, electron transfer number and percentage HO_2_^−^ generated at the disc electrode using N-doped electrocatalysts in 0.10 M KOH on oxygen saturated solution [53] (Figure 4). The ring (Figure 4B) and disc (Figure 4C) currents of N-SAO, N-SAOrGO, N-SAOPF and N-rGOPF electrocatalysts were analysed at 2000 rpm at a scan rate of 10 mV/s. The ORR onset potential measured for these catalysts is displayed in Table 2 and Figure 5A. The results showed the onset potential increased in the order of N-SAOPF (0.98) > N-SAOrGO (0.93 V) > N-SAO (0.87 V) > N-rGOPF (0.78 V). The results show the catalysts which possess macropores demonstrate higher ORR activity compared to N-rGOPF. The ORR onset potential of N-SAOPF showed similar values as the standard Pt/C catalysts (0.98 V).

Conversely, the current density of the catalysts increased in the order of N-SAOrGO (7.89 mAcm^−2^) > N-SAO (6.29 mAcm^−2^) > Pt/C (5.85 mAcm^−2^) > N-SAOPF (5.18 mAcm^−2^) > N-rGOPF (3.99 mAcm^−2^) at 0.00 V (RHE) (Figure 5B and Table 3). Lai et al. [53] previously investigated the active sites of the N-doped nonmetallic catalysts and reported pyridinic nitrogen species improve the onset potentials while the graphitic nitrogen determine the limiting current density. Our results were consistent with the finding of Lai et al. [53]. The N-SAOPF with the higher percentage atomic concentration of pyridinic nitrogen showed the highest positive onset potential compared to other N-doped catalysts. However, it showed low limiting current density due to the lower graphitic N content and nonconductive nature of PF polymer. Conversely, N-SAOrGO showed the highest limiting current density due to the presence of more conductive graphene formed within the composite material as a result of thermal reduction of rGO which facilitate electron charge transfer [26]. Moreover, the presence of higher graphitic N species further increased the current density [53]. The synergetic effect of both conductivity of graphene along with graphitic N species showed higher limiting current density for SAOrGO nearly 65% more than that of standard Pt/C catalyst at 0.00 V (RHE). The Tafel slope of N-SAO, N-SAOPF, N-SAOrGO, N-rGOPF and Pt/C is shown in Figure 5D. The catalysts N-SAOPF and N-SAO shows a Tafel slope of 126.53 mV/dec and 127.53 mV/dec, respectively, which is close to 120 mV/dec. This indicates that either the initial electron transfer step, or the electron transfer step to the *OOH species is the rate limiting step [54]. We also observe that N-SAOrGO and rGOPF has a higher Tafel slope compared to N-SAOPF and N-SAO with Tafel slopes of 146.48 and 185.12 mV/dec, respectively. Therefore, N-SAOPF and N-SAO exhibits better kinetics and can better facilitate electron transfer to the adsorbed intermediates.

The number of electrons transferred and percentage HO_2_^−^ generated in the potential region 0.00–1.15 V are presented in Figure 4D,E, respectively. The comparison of number of electrons transferred (Figure 4C and Table 2) and percentage HO_2_^−^ yield (Figure 4D and Table 2) at 0.5 V revealed N-SAOPF possess the highest electron transfer number (3.62) with the lowest percentage HO_2_^−^ yield (19.02%). According to Liu et al. [49], pyridinic nitrogen provides one *p* electron to the aromatic π system of the carbon matrix to increase the electron-donating capability of carbons adjacent to nitrogen to weaken the O-O bond strength and facilitate the ORR reaction [49]. The highest ORR catalytic activity displayed by N-SAOPF can be attributed to the presence of percentage atomic percentage of pyridinic nitrogen which provided more active sites for oxygen adsorption. Conversely, N-SAOrGO with graphitic nitrogen showed lower catalytic activity for ORR than N-SAOPF. Here, the ORR was performed via 3.38 electrons which associated with a yield of 30.84% HO_2_^−^.

The half-wave potential (E_1/2_) of the catalysts positively increased according to N-SAOPF (0.79 V) > N-SAOrGO (0.66 V) > N-SAO (0.65 V) > N-rGOPF (0.44 V), showing that the ORR performed through both mixed diffusion and kinetic regions where current is controlled by both mass transport and kinetics of electron transfer [55]. The E_1/2_ of the catalysts shifted more negatively compared to the E_1/2_ of the standard Pt/C catalyst (0.88 V). However, among all catalysts E_1/2_ of N-SAOPF shifted more positively in the mixed kinetics and diffusion range region revealing the coverage of high oxygen adsorption. The negative shift of the E_1/2_ of the catalysts can be attributed to their N-C active sites as reported by Liu et al. [56]. However, according to the authors, E_1/2_ can be shifted positively by the addition of Fe-N-C or Co-N-C active sites [56].

The RRDE voltammogram conducted on nondoped catalysts are were also evaluated and shown in Figure S2 and Table S1. The comparison of the mean values of onset potential, current density, number of electrons transferred and percentage of HO_2_^−^ calculated on three samples of doped and nondoped catalysts are shown in Figure 5 and Table S2. The comparison shows onset potential, current density, number of electrons transferred and the percentage of HO_2_^−^ has significantly increased due to N-doping.

**Figure 6:**
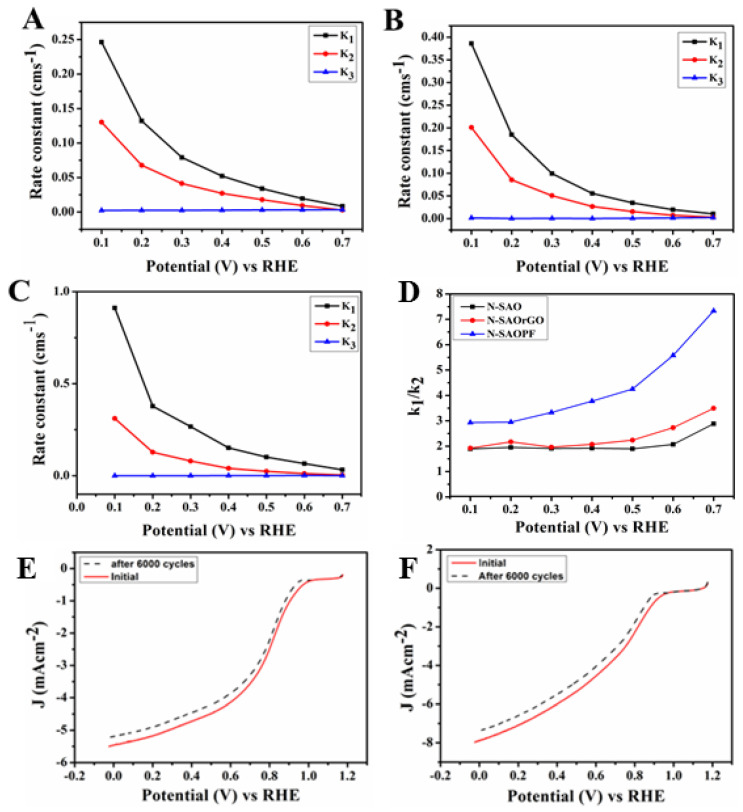
Rate constants of (**A**) N-SAO, (**B**) N-SAOrGO and (**C**) N-SAOPF and (**D**) the ratio of rate constant k_1_/k_2_ for N-SAO, N-SAOrGO and N-SAOPF in the potential range of 0.10–0.70 V. Rotating disc electrode polarisation curves of (**E**) N-SAOPF and (**F**) N-SAOrGO with a scan rate of 10 mVs^−1^ before and after 6000 potential cycles in 0.10 M Oxygen saturated KOH.

To further investigate the electron transfer kinetics of the N-doped catalysts, the scheme suggested by Damjanovic et al. [57] detailed in Supplementary Materials (Scheme S2) was used. The rate constant of N-SAO, N-SAOrGO and N-SAOPF and N-rGOPF calculated based on these equations in the potential region 0.10–0.70 V (Figure 6A–C) shows that the ORR was predominantly driven by a four-electron pathway. The calculated value of the ratio of k_1_/k_2_ presented in Figure 6D showed k_1_/k_2_ > 1 for all catalysts. Among N-SAO, N-SAOrGO and N-SAOPF catalysts, the highest k_1_/k_2_ ratio was observed for N-SAOPF while N-SAO showed the lowest k_1_/k_2_ values. This furthermore reveals that the N-SAOPF with a high percentage atomic concentration of pyridinic nitrogen dominated the ORR catalytic performance. The stability of N-SAOrGO and N-SAOPF was evaluated by cycling the catalysts between 0.0 and 1.15 V at 100 mV s^−1^ in an O_2_ saturated 0.10 M KOH solution. Figure 6E and F shows after 6000 cycles the onset potential of N-SAOrGO and N-SAOPF has shifted 40 and 30 mV negatively showing only a slight deterioration of the catalysts.

These investigations reveal that macropores which are catalytically active for ORR can be synthesised using different composite materials.

The catalysts prepared were compared with similar MCM catalysts and a comparison of electrochemical properties is presented in Table 4. The comparison shows that the onset potential of the N-SAOPF is similar or higher than the other catalysts, while the limiting current density of N-SAOrGO is the highest. However, the number of electrons transferred from N-SAOPF and N-SAOrGO is fewer than the other catalysts. Therefore, this unique procedure adopted to synthesise MCM can be used to synthesise diverse composite materials with SAO to achieve the ultimate electrochemical properties for ORR in the future.

## 4. Conclusions

In this study, we have demonstrated a unique synthesis method to tailor MCMs by a pyrolysis process of SAO using in situ released SO_2_ as a blowing agent. The SO_2_ gas released as a thermal decomposition product of sulphonic groups attached to the SAO has expanded the spherical spherulites of SAO to fabricate MCMs. Macroporous N-doped MCM catalysts, N-SAO, N-SAOrGO and N-SAOPF, showed diverse active sites for oxygen adsorption. N-SAOPF with the highest percentage atomic concentration of pyridinic nitrogen showed the highest onset potential (0.98 V), highest electron transfer number (3.32) and lowest percentage HO_2_^−^ yield (19.02%). Conversely, N-SAOrGO which exhibited more graphitic nitrogen species showed an increased limiting current density (7.89 mVcm^−2^).

Furthermore, this method can be a game changing synthesis process to fabricate new-state-of-art MCM N-doped carbon catalysts with selected active sites for ORR. Electrocatalytically active materials such as metallic nanoparticles, carbon nanotubes (CNTs) and single or few-layer graphene can be used to form composites with SAO to create new catalysts.

## Figures and Tables

**Table 1:** Atomic concentrations of N-doped catalysts.

Element	N-SAO (% At)	N-SAOrGO (%At)	N-SAOPF (%At)
C 1s	87.01	90.53	85.45
N 1s	8.78	4.86	8.89
O 1s	3.96	4.04	4.89
S 2p	0.25	0.57	0.77

**Table 2:** Percentage atomic concentration of nitrogen species present in N-SAO, N-SAOrGO and N-SAOPF.

Nitrogen Species	% Atomic Concentration		
	N-SAO	N-SAOrGO	N-SAOPF
Pyridinic nitrogen	22.24	36.69	42.40
Graphitic nitrogen	77.76	63.31	57.60

**Table 3:** Electrochemical properties of the N-doped SAO and composites of SAO catalysts.

Catalysts	Current Density (mA/cm^2^) at 0.00 V (RHE)	Onset Potential (RHE) (V)	Half-Wave Potential (RHE) (V)	Number of Electrons (n) (0.10–0.80 V) (RHE)	% HO_2_^−^ (0.10–0.80 V) (RHE)
N-SAO	6.29	0.87	0.65	3.23–3.56	38.82–21.86
N-SAOrGO	7.89	0.93	0.66	3.37–3.55	31.88–22.13
N-SAOPF	5.18	0.98	0.79	3.48–3.75	25.67–12.12
N-rGOPF	3.99	0.78	0.44	3.21–2.45	39.28–77.07
Pt/C	5.85	0.98	0.88	3.68–3.98	11.73–9.65

**Table 4:** Comparison of the catalysts with similar MCM materials.

Catalysts	Carbon Material	Onset Potential (RHE)	Limiting Current Density (mAcm^−2^)	Number of Electron (n)	Reference
NPCs	N-doped porous carbon	0.92	5.65	4.03	[58]
N-HPCNS-800	Porous N-doped carbon nanoplates	1.00	6.15	3.82	[59]
3D NPC-800	Biomass derived 3D N-doped porous carbon	0.88	-	3.92	[60]
NPCS-850	N-doped porous carbon sheet	0.98	5.76	3.92	[61]
FePc/CS500	Macroporous hollow nanocarbon	-	5.44	3.99	[62]
N-SAO	Macroporous carbon	0.87	6.29	3.22	This study
N-SAOPE	Macroporous carbon	0.98	5.18	3.62	This study
N-SAOrGO	Macroporous carbon	0.93	7.89	3.38	This study

## Data Availability

MDPI Research Data Policies.

## Supplementary Materials

The following are available online at https://www.mdpi.com/2079-4991/11/1/43/s1. Scheme S1: Schematic representation of custom-build high temperature pulsed-gas sampling equipment, Figure S1: SEM and product images of P-SAOPF and P-SAOrGO, Figure S2: Rotating ring disc voltammograms, number of electrons and %HO_2_^−^ of P-SAO, P-SAOrGO, P-SAOPF and P-rGOPF, Table S1: Electrochemical properties of the pyrolysed aniline oligomers and composites of aniline oligomer catalysts, Table S2: Mean and standard deviations of onset set potential, current density, number of electrons and % HO_2_^−^ of pyrolysed and N-doped carbon materials synthesised, Scheme S2: Proposed model for electrochemical reduction of oxygen.
