# Supplementary material for: A Unique Synthesis of Macroporous N-Doped Carbon Composite Catalyst for Oxygen Reduction Reaction
Source: Nanomaterials (Basel). 2020 Dec 26;11(1):43. doi: 10.3390/nano11010043 (PMC7824199; doi:10.3390/nano11010043)
Supplement: Supplementary file 1 [file nanomaterials-11-00043-s001.pdf]

Supporting information

S1: Thermometric -Mass spectrometer analysis

The thermometric-MS experiment was carried out using a custom-built high temperature pulsed-gas sampling equipment which was coupled with a mass spectrometer for real-time analysis of the gaseous mixture (Scheme S1) [1]. The reaction cell is in contact with a copper block containing a 100 W heater cartridge controlled by a proportional-integral-derivative (PID) temperature controller (CAL Controls, Cal 3300) and K-type thermocouple mounted to the cell. A piezo-resistive manometer (Keller, Leo Record series, 30 bar range) measures the pressure. Pulsed nozzle (Parker, Series 9 Pulse Valve) connected to the cell, which is used to pulse controlled amounts of gas from the reaction cell via a 1/16" stainless steel tube into the vacuum system, comprised of a residual gas analyser (RGA, RGA 200, Stanford Research Systems, USA) for sampling of the gas mixture (*vide infra*). For our analysis, we loaded 10 mg of samples into the reaction cell and ramped for different temperatures including 30 °C to 200 °C and 200 °C–250 °C and 250 °C–275 °C and paused the temperatures at 200 °C, 250 °C and 275 °C to determine any evolved gases.

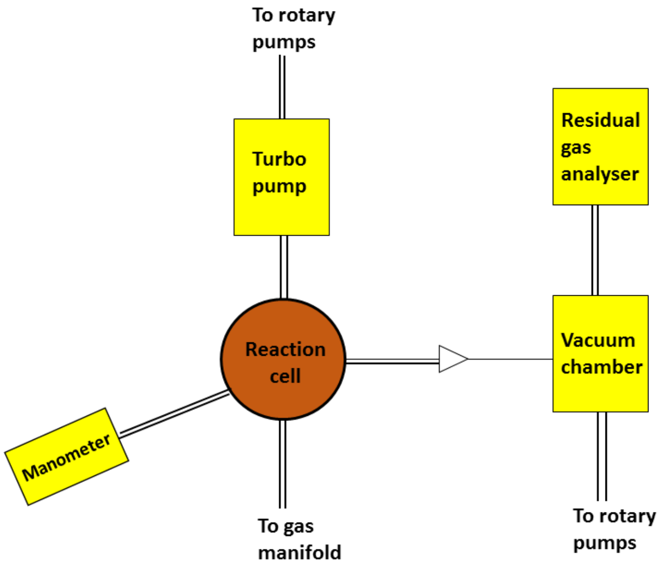

Scheme S1. Schematic representation of custom-build high temperature pulsed-gas sampling equipment.

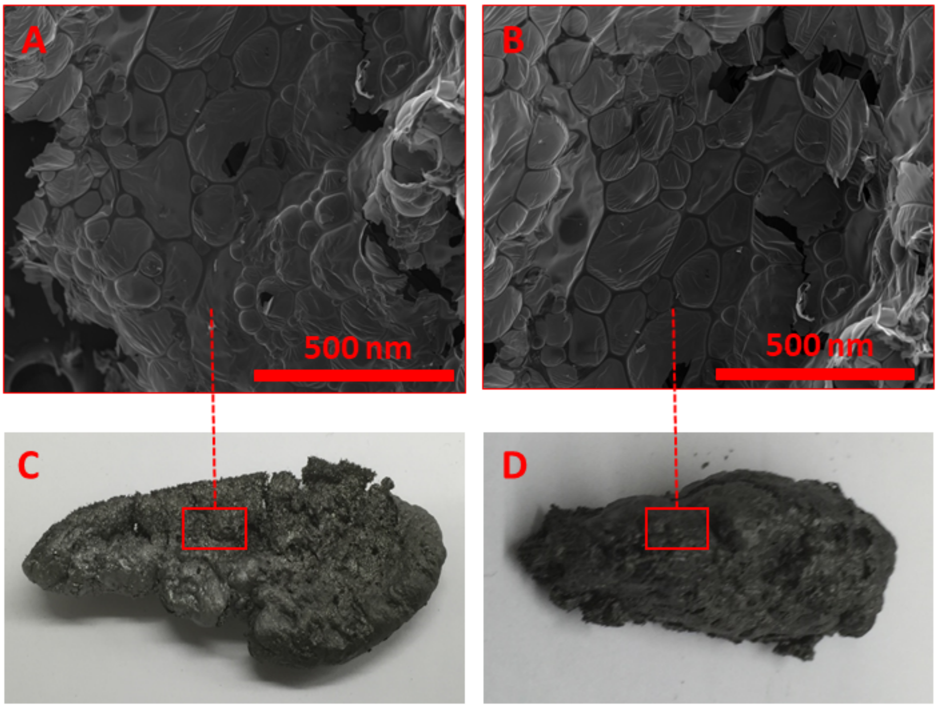

Figure S1: SEM images of (A) P-SAOPF and (B) P-SAOrGO. Synthesised macroporous (C) P-SAOPF and (D) P-SAOrGO.

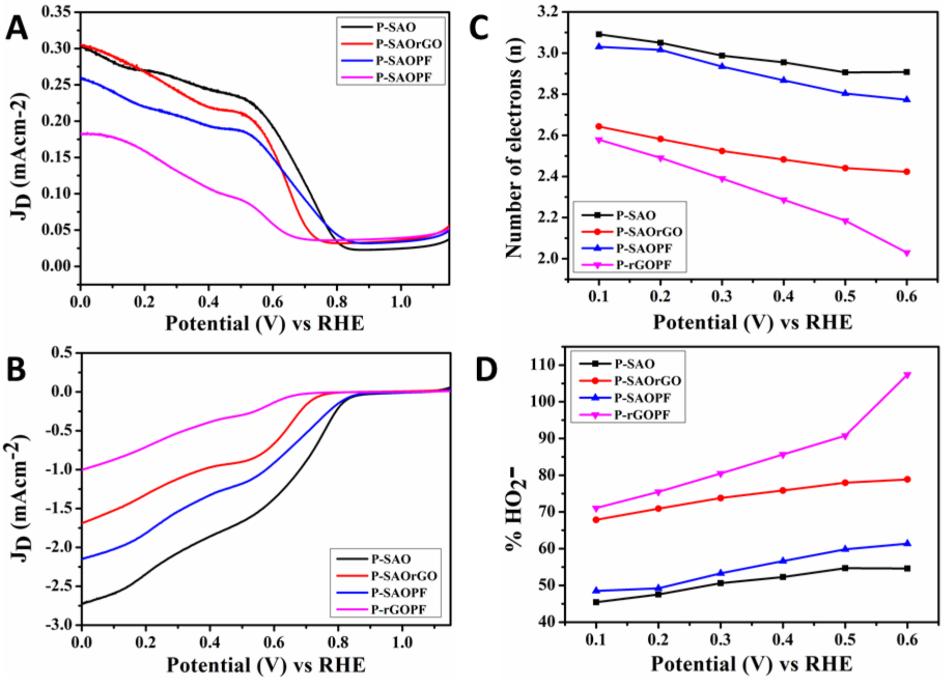

Figure S2. Rotating ring disc voltammograms of (A) ring current, (B) disc current of P-SAO, P-SAOrGO, P-SAOPF and Pt/C electrodes in oxygen saturated 0.1M KOH at 2000 rpm at a scan rate of 10 mV/s. (C) number of electrons and (D) percentage peroxide of P-SAO, P-SAOrGO, P-SAOPF and Pt/C electrodes at various potential calculated according to RRDE data.

Table S1. Electrochemical properties of the pyrolysed aniline oligomers and composite of aniline oligomers catalysts.

Catalysts	Current density (mA/cm^2) at 0.00 V (RHE)	Onset potential (RHE) (V)	Number of electrons (n) (0.10–0.80 V) (RHE)	% HO_2^- (0.10–0.80V) (RHE)
P-SAO	2.73	0.81	3.09–2.90	45.45–54.60
P-SAOrGO	1.69	0.74	2.64–2.42	67.84–78.68
P-SAOPF	2.16	0.84	3.03–2.77	48.50–61.35
P-rGOPF	1.01	0.64	2.57–2.03	71.06–107.40

Table S2: Mean onset set potential, current density, number of electrons and % HO_2^- of pyrolysed and N-doped carbon materials synthesised.

**Onset potential RHE (V)**						
	Mean Pyrolysed	SD	Mean Doped	SD	-	SD
SAO	0.81	0.02	0.87	0.01	-	-
SAOrGO	0.789	0.01	0.93	0.01	-	-
SAOPF	0.84	0.03	0.98	0.01	-	-
rGOPF	0.84	0.01	0.78	0.02	-	-
Pt/C	-	-	-	-	0.98	0.005
**Current density ($mVcm^{-2}$)**						
	Mean Pyrolysed	SD	Mean Doped	SD	-	SD
SAO	2.73	0.06	6.29	0.14	-	-
SAOrGO	1.59	0.10	7.89	0.20	-	-
SAOPF	2.16	0.08	5.18	0.10	-	-
rGOPF	1.01	0.13	3.99	0.11	-	-
Pt/C	-	-	-	-	5.85	0.12
**Number of electrons (n)**						
	Mean Pyrolysed	SD	Mean Doped	SD	-	SD
SAO	2.91	0.01	3.22	0.04	-	-
SAOrGO	2.69	0.13	3.38	0.04	-	-
SAOPF	2.81	0.04	3.62	0.04	-	-
rGOPF	2.19	0.09	3.21	0.05	-	-
Pt/C	-	-	-	-	3.88	0.01
**% HO_2^-**						
	Mean Pyrolysed	SD	Mean Doped	SD	-	SD
SAO	54.69	4.24	39.05	2.98	-	-
SAOrGO	65.37	4.07	30.84	3.05	-	-
SAOPF	59.84	4.10	19.02	2.50	-	-
rGOPF	90.74	4.80	40.19	3.60	-	-
Pt/C	-	-	-	-	10.21	1.30

S2: Electron transfer kinetics

The electron transfer kinetic of the ORR was identified using RRDE voltametry (Scheme S2) [2, 3]. According to Damjanovic [2] *et al.* [2] the electron transfer mechanism follows a direct four-electron pathway via K_1 kinetics (Scheme S1), in which oxygen is diectly reduced to hydroxide anion (OH^-) or could be driven through a two-electron pathway via K_2 kinetics producing peroxide intermediates (HO_2^-), followed by reduction to hydroxide anion (OH^-) through another two electron pathway through K_3 kinetics.

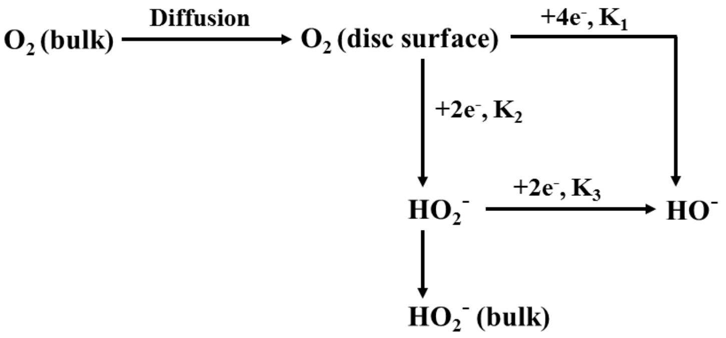

Scheme S2: Proposed model for electrochemical reduction of oxygen proposed by Damjanovic et al. and Hsueh et al.

Hsueh *et al.*[3] suggested a series of equations (3, 4 and 5) to calculate the rate constants K_1, K_2 *and* $K_{3.}$, where Id, Ir, IdL and ω are the disc current, ring current, limiting disc current and the rotation speed, respectively.

$$k_1 = S_1 Z_1 \frac{I_1 N-1}{I_1 N+1} \quad (3)$$

$$k_2 = \frac{2\, S_2 Z_1}{I_1 N+1} \quad (4)$$

$$k_3 = \frac{N S_1 Z_2}{I_1 N+1} \quad (5)$$

Where S_1 and I_1 are the slope and intercept correspond to the I_d / I_r vs $\omega^{-1/2}$ plots and S_2 and is the slope of I_{dL} / I_{dL} – I_d vs $\omega^{-1/2}$ plot. $Z_1 = 0.62 D_{O_2}{}^{2/3} V^{-1/6}$, $Z_2 = 0.62 D_{H_2O_2}{}^{2/3} V^{-1/6}$, $D_{H_2O_2}$ is 6.8 x 10^{-6} cm^2 s^{-1} and N is the collection efficiency [4].

References

[1] Burgun A.; Coghlan C.J.; Huang D.M.; Chen W.; Horike S.; Kitagawa S.; Alvino J.F.; Metha G.F.;. Sumby C.J; Doonan C.J. Mapping-Out Catalytic Processes in a Metal–Organic Framework with Single-Crystal X-ray Crystallography. *Angew. Chem. Int. Ed.* **2017**,56,8412 - 8416.

[2] Damjanovic A.; Genshaw M.A.; Bockris J.O. Distinction between Intermediates Produced in Main and Side Electrodic Reactions, *J. Chem. Phy,* **1966**, 45, 4057-4059.

[3] Hsueh K.L.; Chin D.T.; Srinivasan S. Electrode kinetics of oxygen reduction: A theoretical and experimental analysis of the rotating ring-disc electrode method. *J. Electroanal.Chem. Interfacial Electrochem.* **1983**, 153, 79-95.

[4] Muthukrishnan A.; Nabae Y.; Chang C.W.; Okajima T.; Ohsaka T. A high-performance Fe and nitrogen doped catalyst derived from diazoniapentaphene salt and phenolic resin mixture for oxygen reduction reaction. *Catal.Sci.Technol.* **2015**, 5, 1764-1774.
